# Collagen Bioinks for Bioprinting: A Systematic Review of Hydrogel Properties, Bioprinting Parameters, Protocols, and Bioprinted Structure Characteristics

**DOI:** 10.3390/biomedicines9091137

**Published:** 2021-09-01

**Authors:** Jana Stepanovska, Monika Supova, Karel Hanzalek, Antonin Broz, Roman Matejka

**Affiliations:** 1 Department of Biomedical Technology, Faculty of Biomedical Engineering, Czech Technical University in Prague, Sitna 3105, 272 01 Kladno, Czech Republic; karel.hanzalek@fbmi.cvut.cz (K.H.); roman.matejka@fbmi.cvut.cz (R.M.); 2 Department of Composites and Carbon Materials, Institute of Rock Structure and Mechanics, Czech Academy of Sciences, 182 09 Prague, Czech Republic; supova@irsm.cas.cz; 3 Department of Biomaterials and Tissue Engineering, Institute of Physiology of the Czech Academy of Sciences, Videnska 1083, 142 20 Prague, Czech Republic; antonin.broz@fgu.cas.cz

**Keywords:** bioink, bioprinting, collagen, hydrogel, hydrogel properties, bioprinting parameters

## Abstract

Bioprinting is a modern tool suitable for creating cell scaffolds and tissue or organ carriers from polymers that mimic tissue properties and create a natural environment for cell development. A wide range of polymers, both natural and synthetic, are used, including extracellular matrix and collagen-based polymers. Bioprinting technologies, based on syringe deposition or laser technologies, are optimal tools for creating precise constructs precisely from the combination of collagen hydrogel and cells. This review describes the different stages of bioprinting, from the extraction of collagen hydrogels and bioink preparation, over the parameters of the printing itself, to the final testing of the constructs. This study mainly focuses on the use of physically crosslinked high-concentrated collagen hydrogels, which represents the optimal way to create a biocompatible 3D construct with sufficient stiffness. The cell viability in these gels is mainly influenced by the composition of the bioink and the parameters of the bioprinting process itself (temperature, pressure, cell density, etc.). In addition, a detailed table is included that lists the bioprinting parameters and composition of custom bioinks from current studies focusing on printing collagen gels without the addition of other polymers. Last but not least, our work also tries to refute the often-mentioned fact that highly concentrated collagen hydrogel is not suitable for 3D bioprinting and cell growth and development.

## 1. Introduction

Biofabrication is a rapidly developing field of tissue engineering which combines principles, protocols, and fabrication techniques from biomedicine, engineering, electronics, material science, and cell biology [1]. The aim of this field is the development of three-dimensional (3D) native-like heterogenous complexes with incorporated cells. Bioprinting of polymers allows the generation of complex structures with characteristics similar to biological mass or extracellular matter, which increases the possibility of tissue creation [2]. In this case, the cells and other biologics are deposited in a hydrogel to create a controlled pattern to fabricate living tissues and organs. The cell-laden polymers are called bioinks [3], usually constituted by biomaterials, biochemical molecules, living cells, or any mixture [4]. The process of using bioinks hampers by the need to use a delivery medium for cells which can be deposited into designed shapes acquired from computer-aided design (CAD) models [5]. However, the use of appropriate bioink provides cell-binding sites that are desirable for cell attachment, spreading, growth, and differentiation [6].

In the last two decades, a lot of biomaterials, synthetic and natural origin, were used in the area of tissue engineering. Still, only a few of them have features to create an ideal bioink [7]. The ideal features of polymers allowing bioprinting are bioprintability, high mechanical integrity and stability, insolubility in cell culture medium, biodegradability at a rate appropriate to the regenerating tissue, nontoxicity, non-immunogenicity, and the ability to promote cell adhesion [3]. At the same time, proper mechanical, rheological, chemical, and biological characteristics should be provided [8]. Natural polymers, bio-derived materials present in nature, extracted using physical or chemical methods [9], are used more than synthetic materials, due to their better biological compatibility, while artificially created materials achieve better mechanical properties [10]. Eventually, easy manufacturing and processing, affordability, and commercial availability are essential in choosing the appropriate bioink [3].

The group of natural bioinks is represented by protein-based bioinks, polysaccharides bioinks, and bioinks based on extracellular matrix (ECM) from decellularized tissues (dECM-based) [11]. These ECM-based hydrogels also create a group of physical hydrogels characterized by the reversible nature of crosslinking mechanism and suitability for bioprinting and excellent bioactivity [12]. While collagen is the main structural protein of ECM and has a high affinity for adherent cells [13], collagen hydrogels are widely used in biomedical applications, from testing materials for 3D bioprinting [14,15,16], through general tissue models for in vitro cell studies, drug testing [17,18], to specialized tissue models for osteogenic [19], neural [20], skin [17,21], or epithelium [18] applications, mainly when cell-laden hydrogels are used [22].

Because of good biocompatibility and low immunogenicity, collagen is successfully used in clinical practice [23]. However, low immunogenicity can be obtained only by using high purity collagen solutions when the protein is derived from collagen-containing tissues [23]. The main weaknesses of this material are its low mechanical properties, difficult sterilization (e.g., heat sensitivity, degradation), and the commonly occurring shrinkage (contraction) of collagen scaffolds in response to cellular activity [24,25]. Using decellularized extracellular matrix is associated with the risk of immunological rejection, limiting the possibility of such material in clinical applications [26].

To control material properties, type I collagen was modified with photoactive methacrylate groups, enabling its crosslinking. Such material is marked as ColMA or CMA. Like classical collagen, the hydrogel comprises small randomly oriented fibers and has the same fundamental characteristics, including spontaneous fibrillar self-assembly and enzymatic biodegradability [27]. After irradiation with 365-nm light, the storage modulus increases fivefold. The viability of encapsulated cells is comparable to collagen type I [27].

**Figure 1:**
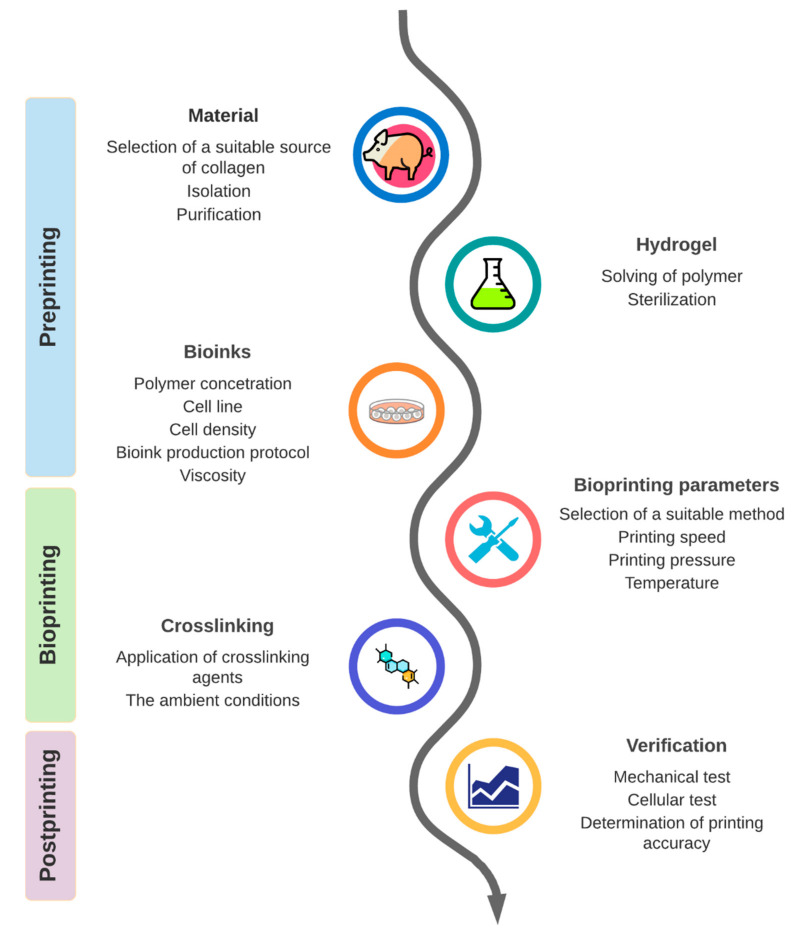
Design and phases of the bioprinting process.

Bioprinting using collagen bioinks has great potential to create an imitation of tissues and manufacture artificial organs in regenerative medicine. The collagen bioprinting process can be divided into several phases (Figure 1), namely preprinting, bioprinting, and postprinting, which are essential for developing a quality 3D construct.

## 2. Methods

A systematic search of studies dealing with 3D bioprinting of collagen bioinks was conducted. The electronic bibliographic databases Web of Science and Scopus has been searched. The literature searches were undertaken in March 2021 and were limited to articles published after 2000, when the first bioprinter system was described [28]. Only original articles were considered. Reviews, overviews, PhD theses, protocols and conference proceedings, studies published in other languages than English, and studies with no available full text were excluded. The search strategy included terms related to the main outcomes–‘collagen’ AND ‘bioprinting’. Originally, the term ‘bioink’ was also searched, but it was found that very few abstracts and keywords contained this word, so it was excluded from the analysis.

The review focuses only on the use of pure collagen hydrogel with cells without additional additives. Therefore, only studies using pure collagen, type I or II, with no other material added, were included in the review. The material could be commercially purchased or laboratory derived, but gels based on decellularized extracellular matrix were not included in the study. Unless collagen was crosslinked by pH and temperature alone, studies using crosslinking agents were not excluded. Other methods of crosslinking (methacrylation with UV irradiation) and printing with scaffolds made of nanofibers or other gels (freeform reversible embedding of suspended hydrogels) were not included.

**Figure 2:**
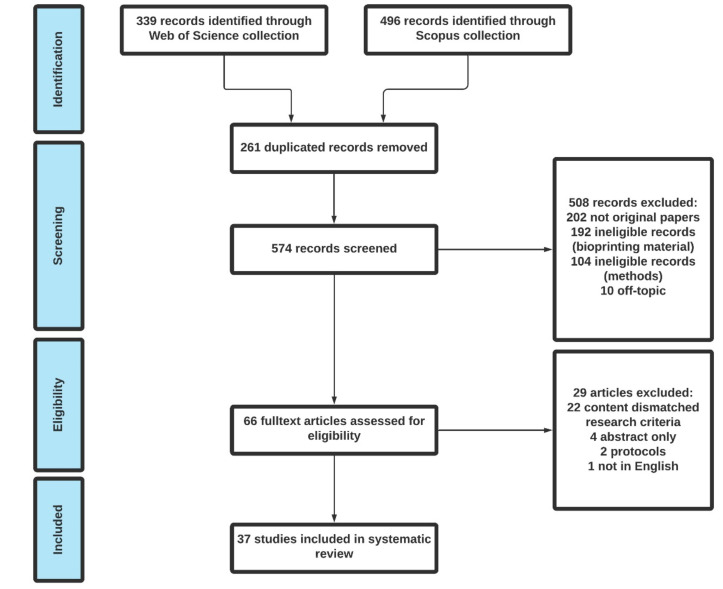
The Prisma workflow diagram of the article search.

All identified titles, keywords and abstracts were imported electronically into the bibliographic database Endnote (version X9.3.3). After excluding duplicates, the titles, keywords and abstracts of the identified articles were screened for inclusion, and then the full text of the selected articles was reviewed by two independent reviewers (J.S. and R.M.) for inclusion or exclusion. The detailed article selection workflow diagram is illustrated in Figure 2.

From the selected studies, basic parameters were summarized in a table (collagen hydrogel composition and concentration, cell density, bioprinting parameters, application). Data extraction was performed independently by two investigators, and in case of disagreement, the article was reanalyzed and discussed to reach a consensus. In our review, the stages of bioprinting are introduced and further described, including an explanation of the most commonly used technologies for 3D printing of collagen bioinks and their parameters. The selected studies according to the above criteria are then listed in a detailed table according to the 3D bioprinting parameters used.

## 3. Results and Discussion

This section is divided into three parts according to the stage of the bioprinting process: preprinting, bioprinting, and postprinting.

### 3.1. Part 1: Preprinting

#### 3.1.1. Origin of Collagen Hydrogels

There are 28 types of collagen superfamily, numbered with Roman numerals from I to XXVIII. They differentiate by the presence of the structural feature triple helix, which can range from most of their structure (96% for collagen I) to less than 10% (XII) [29].

All collagen types differ by tissue distribution and specific biological function. They can also be considered markers of cell differentiation (e.g., collagen XXIV as a marker of osteoblast differentiation and bone formation) [30]. The most abundant are collagen types I–III, constituting 80–90% of total body collagen [31].

Most used bioinks in studies are made from type I collagen, which is the main component in the connective tissues of mammals [32,33].

Collagen comprises two α1(I) chains and one α2(I) chain. The repetitive nature of the amino acid sequence consists of –[Gly (glycine)-X-Y-]n, where X and Y are frequently proline and hydroxyproline residues, allowing the individual left-handed α chains to assemble into right-handed triple helical structures (tropocollagen) with a short, non-helical telopeptide region. Triple helices, with diameters of ~1.5 nm and lengths of 300 nm, are stabilized via intramolecular hydrogen bonding and intramolecular van der Waals interactions. Collagen microfibrils can be defined as an assembly of five individual collagen molecules arranged in parallel along their axial direction via covalent bonds, further associated into fibrils (100–200 nm in diameter), being additionally assembled into collagen fibers [34]. The crosslinks can be both within and between microfibers. Intriguingly, the supertwisted nature of collagen microfibrils is maintained through the non-helical telopeptide regions [35].

**Figure 3:**
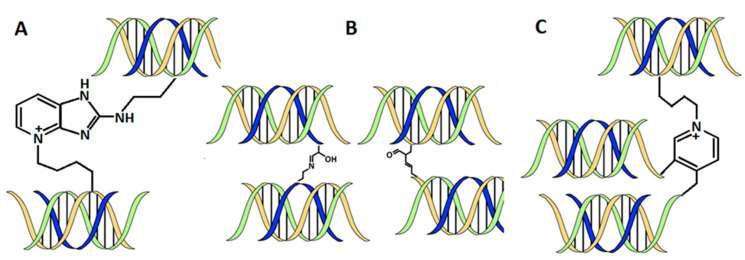
Collagen and its non-enzymatic (**A**) and enzymatic divalent (**B**) and trivalent (**C**) nature crosslinks.

The biosynthesis and maturation of collagen occur via multistep intracellular and extracellular processes, including forming several covalent crosslinks to stabilize collagen structure, confer thermal stability, and provide biochemical properties to tissues [36]. There are two major groups of crosslinks in collagen, namely non-enzymatic and enzymatic [36]. The non-enzymatic crosslinks [37] make up the unselective glycation of lysine residues resulting in the formation of advanced glycation end-products (Figure 3A). Enzymatic crosslinks, which make up most protein–protein crosslinks, exist as a natural product in collagen [38,39]. Intermolecular crosslinking is initiated by the enzymatic oxidative deamination of ε-amino groups of lysine/hydroxylysine residues in the telopeptide domain. Other amines present in these residues can attack aldehydes on both telopeptides and the helical parts to form aldimine-containing crosslinks (Schiff bases). These Schiff bases, known as immature divalent crosslinks in collagen (Figure 3B), are stable under physiological conditions, but they are susceptible to acidic cleavage. Aldimines can spontaneously undergo an Amadori rearrangement to produce ketoimines, such as other types of immature collagen crosslinks [36,40]. Ketoamines are found predominately in bone, cartilage, and tendons. However, they have recently been detected also in the skin [41]. These undergo further reactions to produce mature, chemically more stable trivalent crosslinking structures based on histidine, pyrrole, and pyridinoline (Figure 3C). The crosslinking profiles of collagen in various tissues (skin, bone, tendon, cartilage) are different due to the differences in lysine hydroxylation on telopeptide and helical collagen parts [42]. Other types of collagen crosslinks are rather more tissue-specific than animal species-specific [43].

#### 3.1.2. Collagen Extraction

Collagen is usually obtained from porcine, bovine, murine, or marine connective tissues such as tendons, tails, or skin [44]. A unique extraction process is needed based on the source of collagen and the type of tissue [44]. The amino acid composition of collagen and the position of individual amino acids in α-helix are essential factors that influence the stability of the triple helix [45]. Due to the nature of collagen stabilized by intermolecular interactions and crosslinks, it dissolves very slowly, even in boiling water. The degree of solubility correlates with collagen stability. It depends on the type of biowaste (skin, bone, scales, etc.), the kind of animals (fish, mammals, etc.), and tissue maturity (age). Collagen extraction/isolation can be categorized into chemical hydrolysis and enzymatic hydrolysis [46]. Extraction by chemical hydrolysis is more commonly used. Enzymatic hydrolysis enables preserving the secondary native structures of collagen, but they are more expensive.

Before collagen extraction, pretreatment is used to remove non-collagenous substances, such as lipids, non-collagenous proteins (NP), and glycosaminoglycans (GAGs). Degradation of bonds to saccharides and lipids must be permanent, while disruption of numerous covalent intra- and intermolecular crosslinks in collagen would be temporary.

The degreasing procedure is realized by a detergent or by organic solvents, e.g., diethyl ether. Removal of NP and GAGs can be accomplished by phosphate buffer solutions or low concentration alkali, e.g., 0.05–0.1 M NaOH [47].

The major fibrillar collagens are soluble at low pH, and therefore acids are most commonly used for chemical hydrolysis. Organic acid solutions can solubilize the uncrosslinked collagens and break some interchain cross-linkages of collagen, such as the reducible aldimine condensation crosslinks, leading to further solubilization of collagen during extraction [48]. Therefore, acetic acid is commonly used to extract the collagen. When the pH is further adjusted to around neutral (adding of alkali or by dialysis process), together with physiological temperature, fibril formation occurs spontaneously resulting in collagen reconstruction in vitro [49,50]. Chemical and enzymatic hydrolysis can control obtaining higher yields by variables such as chemical extractors and enzymes, their concentrations, pH, temperature, and processing time [51]. The use of selected animal or vegetable proteolytic enzymes, such as trypsin, chymotrypsin, pepsin, pronase, alcalase, collagenases, bromelain, and papain, permits control of the degree of cleavage of the substrate protein [51]. Isolation by pepsin digestion involves depolymerization of collagen by removing amino and carboxyl-terminal telopeptides that can elicit immunogenic and allergenic reactions [52]. In addition, enzymatic hydrolysis presents some advantages compared with chemical hydrolysis, such as specificity and the control of the degree of hydrolysis [46]. Furthermore, enzymes can be generally employed at very low concentrations, and it is not necessary to remove them from the medium. Despite the high cost of enzymatic hydrolysis, the fact that it results in lower levels of waste, better control of the process, and higher yield justifies the use of enzymes [46].

**Figure 4:**
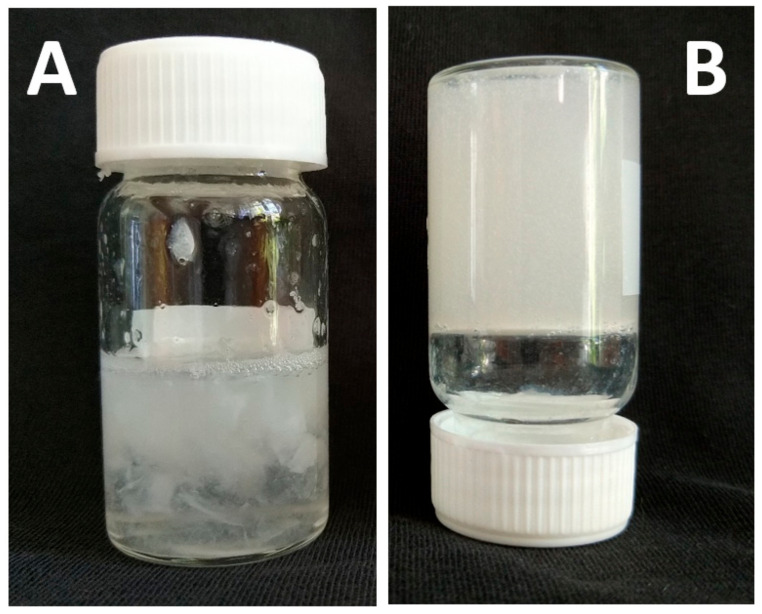
Formation of hydrogel from the same source of collagen however isolated by different way. (**A**) phosphate buffer pre-treatment and extraction by 0.1 M acetic acid; collagen gelled only locally (**B**) ethanol pre-treatment and extraction by 0.02 M acetic acid followed by neutralization by 0.1 M NaOH to pH = 7, collagen precipitate is centrifugated and dissolved again in 0.02 M acetic acid; collagen gelled completely.

The facts mentioned above follow that by extraction/isolation, the original collagen is more or less transformed or altered [53]. Despite the application of the pre-extraction process, isolated collagen may contain minor portions of GAGs, NP, and lipids, which thus become relatively common collagen impurities. The collagen obtained after extraction also has variable levels of telopeptide content, mainly present in the form of monomers with varying amounts of crosslinked components, dimers, trimers, and some higher components [52]. Therefore, the chosen extraction method can affect the final physicochemical properties of the collagen, and consequently the formation of its hydrogel (Figure 4; author’s unpublished results). However, the qualitative and quantitative influence of these components and impurities on the formation and properties of collagen hydrogels has not been systematically investigated and reviewed in the literature.

#### 3.1.3. Collagen Concentration in Hydrogel

The collagen concentration in the hydrogel is most affecting the success of bioprinting. Due to the low viscosity of collagen, other biomaterials must be added to improve its bioprintability. Eventually, the collagen concentration must be increased [54]. Weak mechanical strength and low thermal stability are observed in hydrogels with low collagen concentration. On the other hand, high-concentrated gels report inhomogeneities in structure and worse potential for cell growth [55].

However, only high-density single-component collagen (over 20 mg/mL) bioinks have the potential to create an accurate 3D structure. Using lower concentration is limited to creating 3D prints, which are planar form, maximally 1–2 mm high. Eventually, other hydrogel components or crosslinking agents must be used. Most of the studies (over 90%) are using collagen concentration <5 mg/mL, eventually <10 mg/mL. Such bioinks achieve pure mechanical properties [3,56] and so it is impossible to create a self-standing 3D construct up to 10 mm in height. One option to solve this problem is using a supporting material based on other hydrogel or nanofiber structures. The FRESH method (Freeform reversible embedding of suspended hydrogels) utilizes a support hydrogel as temporary, thermo-reversible support that can be washed away after printing [15]. These materials, e.g., gelatin, temporary support, softer or more fragile materials, are considered biocompatible. Still, during the polymerization process, diffusion of supportive material into collagen can occur and affect cells’ growth encapsulated in the hydrogel. The effect of residues of supportive material on cultivated cells was not yet fully studied.

High-density collagen constructs are considered not always desirable because the increased stiffness of the constructs can affect the ability of cells to store ECM, limit cell proliferation, and limit the ability to differentiate and diffuse waste products [57]. Many original studies and reviews further report that hydrogels with high collagen concentration could restrict cell proliferation [58,59,60,61]. Therefore, it is recommended to use hydrogels with low concentrations but high viscosity to maintain the shape of the 3D constructs [61]. However, only very few studies are available on the viability and proliferation of mammalian cells in dense collagen hydrogels. Still, there is often mentioned the collapse of cells in such gels during cultivation. Although, some studies debunked those concerns. In one study, it was found that fibroblasts cultivated in high-density collagen gels (40 mg/mL) reach high viability over seven days of cultivation [62]. According to the conclusions of our study, the proliferation and viability of cells in high-density hydrogels depend on the composition of bioink, as described in Section 3.1.5.

The collagen concentration is closely linked to the stiffness of collagen hydrogels. The stiffness can also be improved by increasing the storage modulus before extrusion, leading to better printability [63]. It was shown that hydrogels with higher storage modulus than loss modulus are suitable for bioprinting [62]. Except for higher collagen concentration [54,64], the storage modulus can be enlarged by an increase of NaCl concentration in solution [65] and temperature [64].

Most of the research groups performed their experiments on connective tissue-derived collagen hydrogels. There are only a few commercially available single-component collagen hydrogels designated for bioink creation. These are Lifeink (35 mg/mL, Allevi, Advanced Biomatrix, Philadelphia, PA, USA) and Viscoll (80 mg/mL, Imtek, Moscow, Russia). Collagen solutions from Ibidi (Gräfelfing, Germany) and Cellink (Cellink Advanced Biomatrix, Boston, MA, USA) are offered in relatively low concentrations from 3 to 10 mg/mL.

#### 3.1.4. Viscosity and Cell Density

The viscosity of collagen bioinks plays an important role in the printing process. The viscosity of collagen solutions before polymerization increases with collagen concentration [66]. It’s a crucial factor for the choice of bioprinting technology when physical-mechanical properties are the main limitations. The hydrogel is considered printable for extrusion deposition if it has fulfilled the following conditions: (I) the polymer is soluble in a volatile solvent, and (II) the polymer solution has a viscosity in the range 30–6 × 10^7^ mPa∙s [67]. To prevent nozzle clogging, materials with low viscosity (<30 mPa∙s [68]) and low cell density are required in inkjet printing. The risk of clogging in the extrusion dispensers is lower than with inkjet printing. Laser-assisted printing allows printing materials with relatively high viscosity up to 300 mPa∙s and high cell density at a very good resolution [69].

The optimum viscosity should be chosen particularly because of the impaired cell viability during shear stress-induced deformation during deposition [70]. In addition to the viscosity, the nozzle lumen and applied pressure, and indirectly the concentration of collagen in the hydrogel, further contribute to the magnitude of the shear stress [71]. At a high value of shear stress (>10 kPa), the cell viability is reduced to 76% of normal values (<5 kPa, 96%) [61]. The effects of shear stress have implications for cell biology, e.g., in protein signalling and expression [72], maturation of certain cell phenotypes [73], or stem cell differentiation [74].

The overall viscosity is further influenced by cell density in bioink due to the distortion of the fluid flow and the friction between the hydrogel layers and the cell walls [75]. For cell-laden collagen hydrogels, various cell densities are used according to the application, but most studies use densities in the range of 1–20 mil./mL. Some of them report the optimal density range between 1 and 3 mil./mL, when higher cell densities cause clogging of the dispenser due to cell accumulation and sedimentation [76]. On the contrary, values under this range do not promote proper cell growth [17,76]. Cell densities up to 100 mil./mL increased the viscosity and storage moduli before gelation and improved the precision and printability of bioink [63]. Therefore, it is necessary to consider that the cell density and collagen concentration avoid a high total value of viscosity. The cell viability is most affected by the nozzle lumen and hydrogel composition, especially polymer density, but also the location of the cell in the construct has an equally significant impact, when the lowest viability was observed in the cells in the edges of the construct, probably due to the drying of scaffolds [77,78].

#### 3.1.5. Protocols

The collagen bioink preparation protocols used in research papers are usually derived from the instructions of producers of commercial bioinks. The protocols differ in the temperatures of bioink components and the composition itself. All of them agree on the need to keep collagen on ice (2–10 °C) to ensure good printability and prevent premature crosslinking. Allevi [79] and Cellink [80] recommend that other components, including sterile material, be kept at 37 °C to avoid temperature shock of cells. The final mixture of collagen and cell suspension reaches the temperature of about 20 °C during bioprinting. On the other hand, the protocol of Ibidi company [81] recommends all components be kept on ice, including cell suspension, before mixing and printing out. The bioink temperature then does not exceed 10 °C during the printing process.

The composition of the bioinks also varies according to the manufacturer. Allevi only recommended mixing the cell suspension with collagen hydrogel without any additional components [79]. The use of such protocols leads to decreased concentration of media components required for cell development. In Cellink protocol, the collagen hydrogel is first neutralized by NaOH before mixing with the cell suspension [80]. The Ibidi protocol is based on mixing a cell suspension prepared in 1× concentrated medium with a mixture of collagen, 10× concentrated culture media, NaOH to balance pH and distilled water. The final volume of cell suspension in bioink is one third of the volume [81].

**Figure 5:**
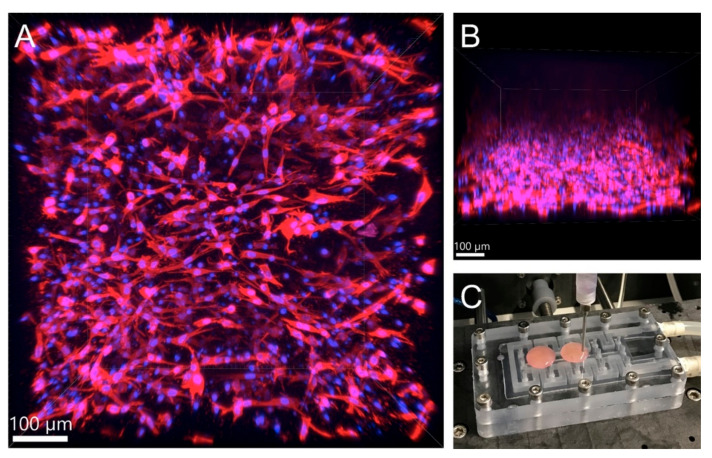
Porcine stromal cells in 3D printed collagen hydrogel (15 mg/mL), substrate coverslip glass, red F-actin, blue cell nuclei. (**A**) 3D image from confocal microscopy. (**B**) side projection from confocal microscopy. (**C**) printing process on substrate fixed using heated vacuum bed. The microscopy image was acquired on Andor Dragonfly spinning disk confocal system with high-speed CMOS camera Zyla 4.2 (Andor Technology Ltd., Belfast, UK) mounted on Leica DMi8 inversed fully mechanized microscope (Leica, Wetzlar, Germany) using 20× lens (NA = 0.75) with water immersion. The wavelengths of the excitation lasers were 405 nm and 561 nm and the transmission characteristics of the emission filters were 450/50 nm and 600/50 nm. Used disk pinhole diameter was 40 µm. The hydrogel samples were photographed in PBS buffer in 35 mm Petri dish with #1.5 glass bottom (0.17 mm thickness, Cellvis, Sunnyvale, CA, USA).

**Figure 6:**
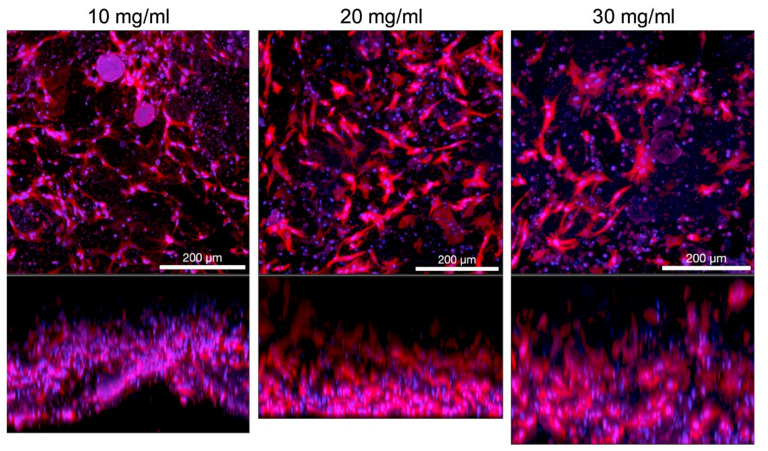
Porcine stromal cells in 3D printed collagen hydrogel with concentrations 10, 20 and 30 mg/mL substrate coverslip glass, red F-actin, blue cell nuclei. Top row shows maximum intensity projection and bottom row show side projection from confocal microscopy. The microscopy images were acquired on Andor Dragonfly spinning disk confocal system with high-speed CMOS camera Zyla 4.2 (Andor Technology Ltd., Belfast, UK) mounted on Leica DMi8 inversed fully mechanized microscope (Leica, Wetzlar, Germany) using 20× lens (NA = 0.75) with water immersion. The wavelengths of the excitation lasers were 405 nm and 561 nm and the transmission characteristics of the emission filters were 450/50 nm and 600/50 nm. Used disk pinhole diameter was 40 µm. The hydrogel samples were photographed in PBS buffer in 35 mm Petri dish with #1.5 glass bottom (0.17 mm thickness, Cellvis, Sunnyvale, CA, USA).

Based on our experience with microextrusion collagen bioprinting, we find the Ibidi protocol to be the most appropriate. The preheating of the cell suspension according to Allevi protocol resulted in non-homogenous bioink, when collagen was separated from the liquid part of the bioink, and the final construction was poorly crosslinked. Cooling of the cell suspension led to creating a homogenous bioink 3D construct with a smaller footprint, which was quickly crosslinked. Similarly, the use of 10× concentrated media in bioink (final concentration 1/15 of final) led to the optimal concentration of growth factors and other media components. The satisfactory cell growth and good viability were then observed even in high concentrated gels (>10 mg/mL) when porcine stromal stem cells derived from adipose tissue were cultivated under static conditions. The protocol of harvesting and their characterisation is described more in our study [82]. Hydrogels were prepared in syringes and 3D printed onto coverslip glass. The print model utilized cylindrical shape with 15 mm diameter and 1.5 mm height. Custom microextruder was used git 17G flat end stainless steel needle. Then the cells were cultivated in static conditions for five days. Process of bioprinting and immunofluorescence-stained F-actin with cell nuclei are illustrated in Figure 5. The cells proliferated in the whole volume of the printed construct. Next, in our experiments, we have compared three different concentrations, i.e., 10, 20, and 30 mg/mL. Figure 6 illustrates different shape of cells based on different concentration of collagen hydrogel. With increasing concentration, the shape of cells changes from elongated spindle shape to more flat shape with multiple filopodias.

### 3.2. Part 2: Bioprinting

#### 3.2.1. Types of Bioprinting Techniques Used for Collagen Bioinks and Their Parameters

**Figure 7:**
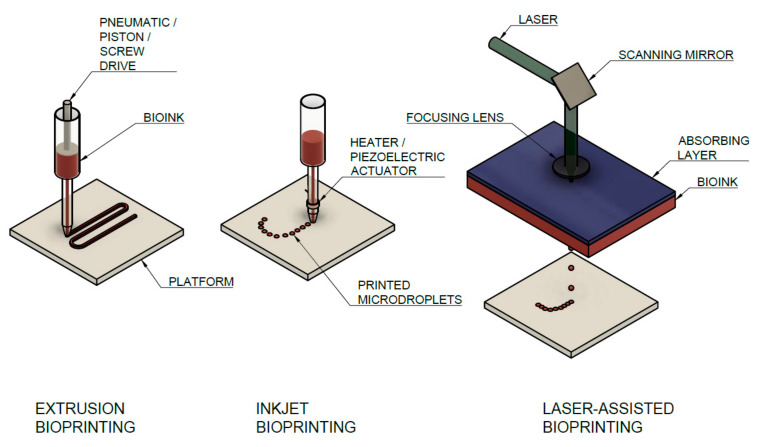
Main additive manufacturing technologies for collagen bioprinting with a description of the main parts of the assemblies.

Collagen is usually printed in preprepared molds or inside other materials that serve as support or are further stabilized after printing by crosslinking [21] due to its low mechanical stability [83]. In general, three main strategies are used for bioprinting of collagen hydrogels, i.e., microextrusion, inkjet, and laser-assisted bioprinting (LaBP) [5]. All methods are shown schematically in Figure 7. All methods are considered as additive biomanufacturing technologies when complex shapes with internal structures without molds or shaping tools are produced [84]. In the first two groups, the material is deposited through the nozzle, when the final structure is created in the resolution which is given: (1) flow rate ratio between the extrudate and printing platform, (2) the nozzle size (3) height of the nozzle above the platform [85]. The own extrusion is driven pneumatically or mechanically by a piston or by a screw [86]. Eventually, the regulation valve is placed at the nozzle to create droplets in the inkjet method. All of these methods are used for collagen bioprinting, and a detailed overview of studies with biotic parameters is given in Table 1.

- Microextrusion Printing

The basic microextrusion method comes from the field of fused deposition modelling when a bioink loaded into a syringe is printed as a filament layer-by-layer using a pneumatic mechanical or electro-magnetic dispensing system [116]. The bioprinting system allows controlled, independent movement in three directions when the material is printed on a platform moving in the y–z plane (another option is the moving of the dispenser on the z-axis), while the printing head moves on the x-axis. For that reason, extrusion-based bioprinting is low cost and widely available, able to deposit high viscosity materials (the range of printable viscosities is 30–6 × 10^7^ mPa∙s [67]), therefore high concentrated collagen hydrogels and high cell density [117,118]. The printing speed and greater deposition allow printing of a relatively large-scaled construct in a short time. On the other hand, the shear stress on the nozzle tip wall and applied pressure cause a significant decrease in the number of living cells when the cell density is high [71]. The printability of 3D structure by microextrusion is further limited by the long crosslinking process and gelation, which cause swelling [56].

The more printing heads allow printing of multicomponent prints, two types of hydrogels, or hydrogel with support scaffold, which enables to create, e.g., tubular constructs [119]. Different custom-made systems [77,78,100] are used for collagen extrusion printing, which allows using up to six dispensing heads [100] or four independent temperature-controlled multi heads, with optical monitoring for each head, crosslinking system, and piezoelectric humidifier [77,78]. The concentric nozzles for printing heterogels based on core/shell configuration were also used [93]. The final precision is affected by many parameters, especially the diameter of nozzle lumen, bioink viscosity, printing speed, humidity, and platform temperature. However, the resolution in the x–y plane is then theoretically limited to hundreds of micrometers, which is considerably lower than the resolution of other methods [120]. The dependence of precision on other parameters was determined experimentally [121], and for syringe extrude methods can be expressed as [121]:

(1) $$a=\frac{{\pi R}_{s}^{4}}{8{\mu v}_{0}h}\;\frac{\mathrm{dp}}{\mathrm{dz}},$$

The line width is a function of the internal radius of the needle tip (R_s_), the height of the polymer pattern (h), the velocity of the substrate concerning the syringe (v_0_), the viscosity of the polymer (μ), and applied pressure gradient (dp/dz).

Bioprinting collagen bioinks is not considered the optimal method for its insufficient mechanical properties when crosslinking collagen after being printed takes considerable time at 37 °C [122]. Extruded collagen must be incubated for 30 min to crosslink. Cell viability is also affected by the time required to bioprint large structures. Bioprinting directly into cell culture media is not performed, leaving the cells exposed to dehydration and nutrient deficiency [123]. Despite this, some studies have adopted this method, e.g., for cell-laden scaffolds for skin substitutes [92] or heterogenous collagen constructs for cartilage tissue engineering [54]. Microextrusion bioprinting enables using a wide array of bioinks based on cell aggregates [124], cell-laden hydrogels [125], microcarriers [126], or components of decellularized matrix [11], which are not printable using other methods of bioprinting.

- Inkjet Printing

Inkjet printer is a noncontact strategy that applies droplets of bioink in a predesigned manner to create the final multilayered pattern. Droplets of defined volume in the range of picolitres are generated by pressure pulses induced by thermal, mechanical, or piezoelectric changes [56]. Droplet volume is then given by the applied pressure and operating/opening actuator time [75]. This allows a fast, low-cost, non-contact method for the bioprinting of bioinks with high resolution. The final 2D or 3D are exact when the spot size resolution is about 50–75 μm [127].

Thermal controlled printing uses the principle of contacting the heating element with the ink for a few microseconds to heat up to 300 °C to produce steam bubbles and bioink drips [128]. The cells in bioinks are exposed to mechanical and thermal strain when short temperature pulses are applied to the bioink (2 μs, 4–10 °C) using thermal control (vaporizing of fluid), or mechanical waves (vibrating piezocrystal) with the frequency of 5–10 kHz [129]. The effect of high-temperature exposure, which originated from droplet formation, was investigated to determine cell viability after printing. Due to its short duration (3 ms), the temperature rise in bioink is marginal with no influence on cell viability [20,130]. For this reason, thermal nozzles are considered as safer for bioinks with mammalian cells because it was shown that electromechanical waves at 15–25 kHz cause cell lysis [131]. The piezocrystal and thermal control are relatively rarely used for collagen bioprinting. Most widely used is microvalve-based bioprinting, when bioink deposition is driven electromagnetically by switching mechanical valves [75], operated by controlled pulse width and frequency to generate the required droplet volume and a given number on a platform [104]. Using this method, bioprinting of thin layers (1–2 μm) with precise positioning and high-throughput printing (about 1000 droplets per second) [17]. Inkjet bioprinting systems are also limited by the viscosity of the bioink (3–30 mPa∙s in the case of a thermo/piezo actuator [68], 1–200 mPa∙s for microvalve controlled printers [75]), so highly concentrated collagen or high cell densities (>1 mil./mL) are almost impossible to print [117].

For the preparation of hydrogels by inkjet bioprinting commercial devices as well as custom-made devices, based on traditional thermal inkjet printers and modified to allow cell deposition [14,20,130], were used. It is possible to print out relatively low collagen concentrations, up to 5 mg/mL [14]. The printability of collagen by inkjet is further affected by pH, when the optimal pH is set to the range 3.5–5, nozzle clogging is observed at higher values [130]. These two parameters further affect the viscosity of the hydrogel, which was lower than 10 mPa∙s in all studies, at cell density 2–5 mil./mL [20]. Applied shear stress, due to high temperature in the nozzle (300 °C), reaches a value up to 10 kPa [61]. The nozzle tends to be clogged due to cell sedimentation [132] and bioink desiccation during the printing process can occur [133].

- Laser-Assisted Bioprinting (LaBP)

Printing by LaBP, transferring bioink from the donor substrate to the recipient substrate by laser beam pulses, focused by lenses, enables the creation of structures near the scale of a single cell [111]. LaBP is based on laser-induced forward transfer (LIFT) when a pulsed laser source is used to deposit a droplet of an organic component (bioink) on a receiving substrate [111]. The energy created by irradiating the absorber layer with the laser beam causes the layer to evaporate, creating pressure that ejects droplets of bioink onto the platform [75]. This is a very precise method with a resolution of tens of μm, based on applied energy, pulse time, and bioink viscosity [134]. Laser beam pulses create precise patterns on the energy-absorbing layer on the top of the donor substrate. The size of bioink droplets then depends on the magnitude of the absorbed energy. The absence of a nozzle precludes clogging of the nozzle, which is common in other printing methods [117], additionally high cell densities (>10 mil./mL [75]) and relatively high viscosities (up to 300 mPa∙s) are possible to print. However, the use of this method is limited by the high cost because of the high price of the printing system, which is not widespread. The thin bioink layer tends to dry up as well as the printed droplets of small volumes, if this is not prevented, e.g., by water flooding [135]. The bioink layer may be further contaminated by residues from the absorbing layer during laser irradiation [136]. Collagen bioinks, carried out especially by laser-assisted bioprinting, are very often used for skin tissue regeneration [109,110].

Stereolithography is often counted among the light-induced methods that include laser assisted bioprinting. The stereolithography method uses light to solidify the hydrogel layer-by-layer. But that method is badly applicable for bioink printing because of the time duration, so it is not used for collagen bioprinting at all [137].

#### 3.2.2. Temperature

For optimal bioprinting, it is necessary to maintain the temperature of the hydrogel and the platform. The temperature of the cartridge is closely related to the reaction kinetics. When self-assembly of collagen molecules can occur at a higher temperature, the viscosity of the hydrogel and the shear stress are affected, as well as, indirectly, cell viability. During collagen gelation, a lag phase exists, where the primary aggregates of molecules are nucleated followed by microfibrillar aggregation (coil-to-helix transition) induced by changes in ionic strength, pH and by raising temperature up to 37 °C until the equilibrium is reached [50]. The gelation process of collagen is thermo-reversible. It melts by lowering the temperature, and gelling occurs by raising the temperature [50].

Most studies are in agreement in that, to avoid premature crosslinking, collagen handling should be carried out at temperatures of 2–10 °C. On the other hand, only a few studies report the temperature of both bioink components, the hydrogel and the cell suspension. The mixture of cold collagen and relatively warm (about 30 °C) cell suspension results in the achievement of physiological values of temperature and pH (6.5–8.5 or 20–37 °C), and fibrillogenesis of collagen molecules directly in the dispenser can occur [50], leading to nozzle clogging. Therefore, it is preferable to prepare a cell suspension with relatively cold media components (10 °C), which is a temperature that will not harm the cells in the short term.

Maintaining the temperature of the platform, where bioink becomes stiff, can prevent the thermal shock of cells and improve cell viability when 37 °C is considered optimal for cell viability [138]. The use of lower temperatures, e.g., 20–26 °C showed inhibition of cell inclusion within the hydrogel [138]. The collagen polymerization under low temperatures (<19 °C) produces the optimal pore size for cell proliferation [139]. Similarly, another study showed that polymerization at room temperature leads to the formation of microchannels and lower hydrogel degradation compared to 37 °C [140]. The bioink is often maintained in the ice-cold temperature range and heated directly on the platform to a physically relevant temperature range [123]. For this reason, the configuration of cooling of the hydrogel stack and dispensing system with nozzle up to 10 °C and heating of the platform with a temperature range of 35–37 °C is used in many studies [94]. A recent study showed that 3D printing of neutralized collagen solutions on a heated platform is not successful unless the concentration of the collagen solution is greater than 7.5 mg/mL [54].

Moreover, collagen is a poor bioink with variable viscosity and elastic modulus throughout the printing process due to its time-sensitive and temperature-sensitive crosslinking. To overcome this problem, Kim et al. [141] used a 3D plotting system coupled with a cryogenic refrigeration system to fabricate a 3D collagen scaffold with designed pore structures and high porosity. However, the fabricated 3D collagen scaffolds occasionally collapsed due to weak adhesion between collagen strands.

#### 3.2.3. Printing Pressure and Nozzle Diameter

The printability of syringe-based systems depends on the printing pressure, which provides a force for bioink extrusion and droplet formation when the minimal pressure value is given mainly by the viscosity of bioink. If the pressure is lower than the minimum printing pressure, large droplets start to accumulate at the nozzle opening because the force to overcome the surface tension of the liquid is insufficient. Conversely, excessive printing pressure would lead to the formation of randomly distributed microdroplets [75]. Printing pressure has a profound effect on cell behavior. Indeed, it was found that cells exposed to printing pressures below 50 kPa showed no harmful short- or long-term damage [142]. However, this limit is also influenced by other parameters, especially the nozzle width, as pressures higher than 1 atmosphere are applied in many studies, and the cell viability is over 90% [142]. Interestingly, the applied pressure does not depend on the type of crosslinking, whether the collagen bioinks are crosslinked by temperature and pH after printing, or various crosslinking agents are added directly to the hydrogel. Nozzle size also has a much greater effect on the resulting droplet size than the applied pressure, where it has been shown that a pressure change in the range of 15–40 kPa has no influence on the droplet diameter [143]. Nozzle size is also a major factor in overall resolution, with a smaller diameter being possible to achieve higher precision, but it is also more difficult to control and more easily clogged [143].

#### 3.2.4. Printing Speed

The time consumption depends on printing technology. When the inkjet technology is considered as lowest, the extrusion bioprinting as the longest, and laser-assisted processes are temporarily in the middle. In general, collagen structures are limited by low mechanical strength and slow forming speed, to which the printing speed must be adapted [144]. The speed of the nozzle extruding the collagen bioink is very relative to the density of collagen in the hydrogel, but it can be considered that a higher speed leads to a more accurate print [62], but the printed structure may not be consistent. The speed of extrusion is given by the speed of screw rotation/piston or applied pressure and nozzle diameter. For these reasons, the speed cannot be precisely controlled. The speed of inkjet-based printers is given by the switching rate of the deposition actuator when the throughput rate is up to 30 kHz (piezocrystals) [145], 1 kHz (microvalves) [17]. However, the real value of inkjet printers with piezocrystal actuator is lower than 500 Hz due to pressure fluctuation within the print head at higher frequencies [143], while microvalve control allows switching up to 1 kHz. Laser-based printers allow droplet deposition with frequencies below 20 Hz [146].

#### 3.2.5. Crosslinking Methods

The isolated collagen, due to the alteration of natural crosslinks and assembly structure by neutral salt, acid, alkali, or proteases during the extraction process, thus exhibits poor thermal stability, mechanical strength, as well as water and enzymatic resistance [147]. To increase their strength and enzyme resistance, and to maintain their stability during implantation, especially for long-term applications, collagenous matrices are usually stabilized by crosslinking. The natural crosslinking pathway of collagen does not occur in vitro, and therefore chemical, physical (artificial), and enzymatic in vitro crosslinking methods have been proposed over the years. However, preclinical and clinical data indicate that crosslinking methods may have adverse effects on host response, especially when potent crosslinking methods are employed [147]. The outcome of crosslinking depends upon the type of crosslinker and the extent of crosslinking. Therefore, some optimization is needed to achieve the desired outcome [148].

Generally, there are chemical and physical methods for collagen crosslinking. The first requires a chemical agent that can interact with collagen through amino and carboxylic functional groups, and which leads to the formation of crosslinks between individual collagen molecules. The most used are aldehydes and carbodiimides, such as glutaraldehyde (GTA) and 1-Ethyl-3-(3-dimethylaminopropyl) carbodiimide (EDC), and N-hydroxysuccinimide (NHS) [149]. GTA-protein crosslinks are formed through the reaction of ε-amine groups of lysine or hydroxylysine residues in collagen with the aldehyde group of GA [149].

GTA is widely used in biomedical engineering because it is inexpensive, easily available, high soluble, rapid crosslinking, and its concentration affects the mechanical and thermal properties of the final hydrogel [150]. However, poor biocompatibility and induction of apoptosis has been observed [151].

EDC/NHS induces the formation of a covalent bond between carboxylic groups of aspartic and glutamic acid in collagen, it plays only such as an activation agent for two collagen molecules without any linkers [152]. Other possibilities are crosslinking with the using of biological molecules such as genipin (GNP) and di-catechol nordihydroguaiaretic acid (NDGA). GNP is a nontoxic biocompatible crosslinker obtained from the iridoid glucoside of Gardenia jasminoides [153]. It is known that GNP reacts with the free NH_2_ groups of Lys, Hyl, and Arg in collagen, forming monomeric/oligomeric bridges [154]. As a consequence, long bridges can link particular macromolecules, which significantly can change the mechanical properties and thermal stability of the composite [153]. NDGA is an antioxidant isolated from the creosote bush. The exact mechanism of NDGA crosslinking is not yet clear, however it seems that NDGA does not crosslink. Rather, it encapsulates collagen fibrils. NDGA molecules are capable of crosslinking each other at each end forming a highly crosslinked polymer which “covers” the collagen fibres and thus prevents collagen degradation [155].

Special interest get natural compounds, such as carbohydrate present in different vegetables, fruits, and honey because they could act as chemical agents for collagen crosslinking via non-enzymatic glycation or Maillard reaction.

Enzymatic-induced crosslinking was introduced in an attempt to overcome some problems with toxicity and biological incompatibility. The oxidative enzymes tyrosinase and laccase [156], as well as acyltransferase, transglutaminase [157], and lysyl oxidase [158], are capable of creating covalent crosslinks in proteinaceous substrates. In addition, transglutaminase and lysyl oxidase do not change the morphology of collagen fibrils and interact in a similar way as naturally occurs in mammalian cells.

**Figure 8:**
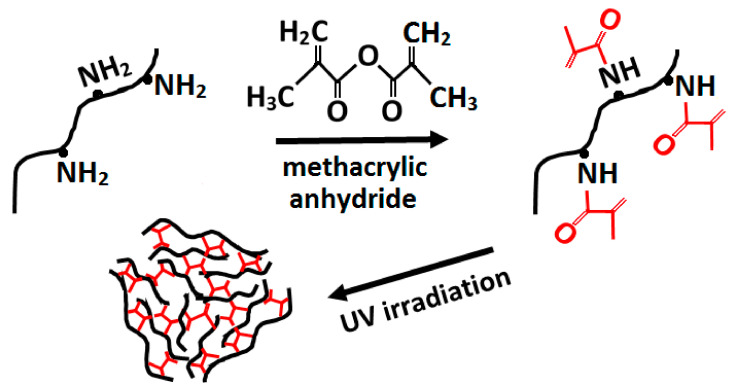
Modification of amino groups in gelatine/collagen with methacrylic anhydride.

Other possibilities for elimination of adding of a potentially toxic agent are physical crosslinking methods including heating, drying, and irradiation which also provides sterilization [159]. Thermal crosslinking of collagen (>90 °C) under vacuum conditions enables the releasing of water molecules and the formation of amide bonds between collagens without any side products generation [160]. However, elevated temperature may cause denaturation [161]. These structure changes may decrease inflammation and increase cellular attachment in vivo [162]. The UV-induced crosslinking reaction is more rapid and effective than thermal treatment. The formation of free radicals on the aromatic groups of tyrosine and phenylalanine enables them to react with others. The novel approach involves the reaction between photo-activatable reagents, such as riboflavin [163] or EDC/NHS [164] and UV light to produce intra- and intermolecular links within the collagen fibres. Prolonged exposure to UV-rays can also cause collagen denaturation and it can be minimized by performing irradiation in oxygen-poor environments [165]. However, UV irradiation only modifies the surface rather than the bulk of the collagen [166]. Crosslinking of gelatine and collagen by the UV-irradiation method involves the modification of amino groups of gelatine and collagen, commonly by methacrylic anhydride (Figure 8) [167]. In comparison to DHT-crosslinking, UV-crosslinking decreased the shrinkage temperature of collagen fibres and increased their solubility in a solution containing enzymes [168].

Crosslinking of collagen chains affects the material mechanical parameters, which depend on the degree of crosslinking and the absence of crystallinity [169]. The swelling of collagen under physiological conditions makes it an ideal material for use as bioink [170]. After physically or chemically crosslinking, an insoluble 3D structure is created, which enables the immobilization and release of active agents and biomolecules. Because of the high water content, the hydrogel mimics natural tissue [170].

The nature of crosslinking bonds leads to differences in microstructure, diffusion, and mechanical differences. Covalently crosslinked collagen has a denser and stronger network but is less permeable [171].

### 3.3. Part 3: Postprinting

#### 3.3.1. Mechanical Tests

The hydrogel stiffness has a large effect on cell morphology, intracellular signalling, and therefore on stem cell differentiation and cell migration [172]. Spatial structures are natural environments for cell development, where the stiffness of the gel matches the corresponding tissue properties in vivo to achieve physiological cell behaviour. The hydrogel network made of individual filaments embedded in aqueous solution mainly contributes to the hydrogel mechanical properties [173]. The values of elastic modulus vary across the type of tissue, from 0.5 kPa (adipose tissue), units of kPa (soft tissues–brain, kidney), tens (heart, intestine), to dozens (bone) and thousands (cartilage) of kPa [174]. Cancer and other diseases are characterized by a relationship between tissue elasticity, cell signalling, and response. Specifically, increased ECM stiffness along with increased collagen concentration is a hallmark of many tumours [175].

Mechanical properties of biological tissues refer to the response of tissues to three types of deformation: tension, compression, and shear. A detailed review of the mechanical test methodology for each deformation mode, including method advantages and limitations, is described in the publication of Chandran et al. [176]. Collagen hydrogels, like soft tissues, are macroscopically incompressible nonlinear viscoelastic materials whose mechanical properties depend on both the applied strain and time [173]. Thus, the values in the literature often vary because the measurements were performed under different ambient conditions [177], different gel preparations and collagen sources were used. Moreover, some of the variables, such as the modulus of elasticity, may be given in tension or in compression [138]. If only a single value is given in the study, an implicit approximation of linear elasticity has been obtained. However, if we compare the collagen hydrogel to a real tissue, we can assume a linear behavior of the material, as most tissue types undergo relatively small deformations under physiological conditions [178].

Collagen forms physical hydrogels with poor mechanical properties unless further crosslinking is performed [179]. This statement generally agrees with other studies comparing the mechanical properties of collagen hydrogels polymerized by temperature and pH adjustment vs. using chemical and enzymatical crosslinking agents. The number of fiber cross-links, formed by physical, chemical, or enzymatic cross-linking, affects the resulting mechanical properties [180]. The physical crosslinks, i.e., the entanglement and attraction of the chains, are considered weaker than the chemical bonds [181]. In the study of Valero et al., physically and enzymatically (adding transglutaminase (TG2)) crosslinked hydrogels showed similar elastic modulus for collagen concentrations ranging from 1.5 to 6 mg/mL [173]. The shear modulus of collagen hydrogels is comparable to collagen with added TG2, the polymerization process occurred earlier. The addition of TG2 to the gels avoids the softening phase caused by the wavy nature of the collagen fibres [182]. For gels crosslinked physically and enzymatically, the storage modulus (G′) was one order of magnitude higher than the loss modulus (G″). Thus, both of them are considered as a viscoelastic solid [173]. Similar results were obtained in the study of Nyambat et al., where collagen hydrogels crosslinked by genipin showed increased stiffness and complex viscosity, while the cell morphology and viability of incorporated adipose-derived stem cells were not significant [183].

Nevertheless, the comparison of crosslinking methods has not been deeply studied in other works. Measuring of the rheology of collagen-based scaffolds with concentrations 5, 10, and 20 mg/mL, crosslinked by pH and temperature, showed a significant increase of elastic and viscous moduli, especially for 20 mg/mL concentration [184]. The values of 20 mg/mL were three orders of magnitude higher than lower concentrations (0.13, 0.4 vs. 144 Pa) [184]. While such values are still lower than those of biological tissue (500 Pa for adipose tissue), they demonstrate that higher collagen concentrations in the hydrogel are a potential route to create a suitable bioink for 3D printing. This is evidenced by a study in which the mechanical properties of collagen bioinks prepared from Viscoll (Viscoll, Imtek Ltd., Russia) were measured, where the final cell concentration was 0.5 mil./mL and the collagen concentration in the hydrogel was 20, 30, and 40 mg/mL (before mixing with cell suspension and other additives) [62]. The modulus of elasticity of these bioinks was then 600, 750, and 900 Pa [62]. These materials were then successfully used to create 3D constructs, where the stiffest gel was used to create a 6 mm cube. Cell viability after seven days of culture was at least 85% for all gel concentrations [62]. The use of highly concentrated gels is thus a suitable alternative for creating 3D prints of cells and tissues. Although chemical and other methods can increase the overall stiffness of the material, their use is associated with the risk of cytotoxicity and lower cell proliferation [180,182]. The higher collagen concentration also leads to higher fiber density with their lower diameter [179]. Gels with higher collagen concentration are also stiffer and polymerization occurs more quickly [173]. The addition of cells to the hydrogel leads to an increase in the storage modulus and viscosity of the collagen bioinks, but also to a reduced rate of gelation. However, the resulting construct achieves a smaller construct footprint, higher resolution, and smaller line width. Thus, the printability of the bioink is increased [87].

However, the studies agree with the following conclusions, independent of the measurement parameters:

- The origin and preparation of the collagen solution has an influence on its mechanical properties [185].
- The collagen concentration [185,186,187] as well as the pH [187] of the solution, are positively correlated with the elastic and shear modulus of the hydrogel.
- To increase storage modulus, the collagen concentration [62] or cell density must be increased [87].
- The mechanical properties can be naturally enhanced using crosslinking agents [188].
- The more concentrated collagen solutions reduce a gelation time [66].

#### 3.3.2. Viability

Whether due to inappropriate properties of the hydrogel or inappropriately chosen bioprinting parameters, apoptosis or necrosis may occur. Therefore, the assessment of cell viability is useful to determine the suitability and efficiency of the method. Typically, the quantification of dead and live cells is based on fluorescent dyes and measuring using flow cytometry or fluorescent microscopy, which is used more often in hydrogels. The second method is cell counting from confocal images of different hydrogel layers, where the nuclei and cytoskeleton of cells are stained, but it is impossible to distinguish between living and dead cells [189].

There are certain limitations to printing hydrogels that lead to a reduction in cell survival rate. To prevent a decrease in cell viability in the printed gel, it is necessary to prevent a local increase in pH, which could be caused, for example, by the release of alkaline ions [190]. Using syringe-based methods, the main factors affecting cell viability are the width of the nozzle and the pressure applied to the cells [191]. In the LaBP method, the cell survival rate depends on the thickness of the hydrogel coating [192] in addition to the laser power [193].

Studies are conflicting on whether collagen concentration affects cell viability. Some of them show a negative correlation with viability, in low-density collagens being around 95% and decreasing to 80% in collagens up to 5 mg/mL [194]. In another study, collagen gel concentration had no effect on cell viability even with highly concentrated hydrogels (12.5–17.5 mg/mL), with viability ranging between 90% and 95% after 11 days [194].

The density of cells in the gel is recommended in the range of 1–3 mil./mL. At lower levels, intercellular contact is minimal, and thus growth and proliferation are restricted, resulting in lower viability [17]. Higher cell concentrations have not been shown to have a negative effect on cell survival but are a limitation for the bioprint itself, as discussed in previous chapters.

## 4. Conclusions

Bioprinting has undergone rapid development in recent years and collagen bioinks appear to be a promising means of creating 3D constructs. Due to the wide range of possibilities for the composition of the hydrogel itself, the choice of crosslinking method, and the variability of collagen and cell density, the applications are broad, ranging from in-vitro studies, drug testing, to the creation of tissue and basic organ models. The easy availability of collagen, where it can be extracted relatively easily from the skin and connective tissues of organisms, also contributes to its use. Our review has shown that it is possible to use pure collagen bioink without additional additives supporting hydrogel stiffness to bioprint relatively strong 3D structures. However, the successful printing of such structures requires a high concentration of collagen in the hydrogel, low temperature of bioink components, and an optimal bioink composition for cell growth and proliferation. Although the physically cross-linked hydrogel has inferior mechanical properties, the use of such a bioink precludes inferior cell proliferation and viability due to the possible cytotoxicity of the added components. Therefore, current research should focus on the growth, migration, and differentiation of cells in highly concentrated collagen hydrogels, as these gels do not need to be further modified to increase mechanical strength and are thus an optimal route to the creation of constructs that are completely natural to the cells and the organism.

## Figures and Tables

**Table 1:** Bioprinting parameters and other conditions of studies dealing with bioprinting of collagen bioinks. PBS—Phosphate Buffered Saline; DMEM—Dulbecco’s Modified Eagle Medium; Ham’s—F-12 Nutrient Mixture; HEPES—4-(2-hydroxyethyl)-1-piperazineethanesulfonic acid; EDTA—ethylenediaminetetraacetic acid; TBS—Tris-buffered saline. 10x—10x concentrated solution, 1x—1x concentrated solution; n.s.—the parameters were not specified by authors of study;—parameter not monitored.

Method	Bioink Components	Collagen Concentration (mg/mL)	Cell Density (mil./mL)	Crosslinking Agent	Temperature (°C)			Pressure (kPa)	Nozzle Diameter (µm)	Actuator	Power (μJ)	Droplet Volume (nL)	Application	Reference
					Hydrogel	Nozzle	Platform							
Extrusion	1× PBS, 10× PBS, NaOH	4, 8, 12	10	riboflavin	On ice	n.s.	37	n.s.	250	-	-	-	Measuring print accuracy and rheological properties with and without riboflavin crosslinking	[63]
Extrusion	1× PBS, 10× PBS, NaOH	8	0.5, 10, 25, 100	n.s.	On ice	n.s.	37	n.s.	250	-	-	-	The effects of incorporating cells on the rheology and printability of collagen bioinks	[87]
Extrusion	10× DMEM, buffer (NaHCO_3_, HEPES, NaOH, H_2_O)	3	20/30	T, pH	On ice	n.s.	n.s.	n.s.	200, 250, 400, 610, 840	-	-	-	Heterogenous tissue structure fabrication	[88]
Extrusion	n.s.	3, 6	10	Pluronic F127	On ice	37	37	50–85	1000	-	-	-	Optimization of bioprinting time and extrusion profile to fabricate 3D collagen constructs	[89]
Extrusion	n.s.	4	2	Tannic acid	On ice	30	30	140–600	310	-	-	-	Study of metabolic activity, cell viability and proliferation of cell-laden collagen structures	[90]
Extrusion	10× DMEM	5	5	Tannic acid	n.s.	5-10	35–37	220	150	-	-	-	The effect of crosslinking agent on physical properties and cellular activity	[91]
Extrusion	4× DMEM, NaOH	3	10-20	T, pH	10	10	n.s.	12/10	90/250	-	-	-	The use of 3D direct-write cell deposition system to construct spatially organized viable structures	[77]
Extrusion	n.s.	3	0.1, 1, 10	T, pH	7	7	no heat or 29–31	14, 28, 36, 60, 120	90, 250	-	-	-	Evaluating the use of a direct-write, 3D bioassembly tool capable of extruding cells and matrix into spatially organized 3D constructs	[78]
Extrusion	PBS, 10× PBS, NaOH	12, 15, 17.5	10	n.s.	n.s.	n.s.	37	n.s.	n.s.	-	-	-	Developing and evaluating a method of printing soft tissue implants with high-density collagen hydrogels using a commercially available 3D printer	[54]
Extrusion	10× DMEM	n.s.	0.5, 3.2	T, pH	n.s.	n.s.	25–60	150	180	-	-	-	Fabrication of 3D cell-laden scaffolds for better skin tissue regeneration	[92]
Extrusion	n.s.	2	5, 6.7	T, pH	n.s.	n.s.	32	12	n.s.	-	-	-	Developing multilayered cell-laden mesh structure and a collagen-based cell-laden bioink	[93]
Extrusion	10× DMEM	3, 5, 7	1	genipin solution	10	10	35	110–300	310	-	-	-	A printing strategy with optimal condition including a safe cross-linking procedure for obtaining a 3D porous cell-block composed of a biocompatible collagen bioink	[94]
Extrusion	10× HAM-F12, NaOH, NaHCO_3_, HEPES	3.5	1.75	T, pH	On ice	n.s.	n.s.	41	n.s.	-	-	-	The fabrication of an implantable multilayered vascularized bioengineered skin graft using 3D bioprinting	[95]
Extrusion	10× DMEM, NaOH	3	1.6	T, pH	n.s.	n.s.	n.s.	n.s.	n.s.	-	-	-	The structure and stiffness of printable hydrogel matrices under different conditions	[96]
Extrusion	NaOH	10	5, 10, 30	T, pH	On ice	20	n.s.	n.s.	250	-	-	-	Collagen type II hydrogel/chondrocyte constructs fabricated using a bioprinter with three different total cell seeding densities in collagen type II pre-gel	[97]
Inkjet	10× DMEM, NaOH	3.3	n.s.	n.s.	On ice	n.s.	n.s.	n.s.	n.s.	piezocrystal	-	15	Using of thermosensitive gels for generating 3D constructs	[98]
Inkjet	PBS	1	0.15	T, pH	On ice	n.s.	n.s.	n.s.	20-30	piezocrystal	-	0.01–0.02	Applying high-throughput inkjet printing to control cellular attachment and proliferation by precise, automated deposition of collagen	[14]
Inkjet	n.s.	0.5	n.s.	n.s.	n.s.	n.s.	n.s.	n.s.	n.s.	piezocrystal	-	n.s.	Preparation a cancer microtissue array in a multi-well format by continuous deposition of collagen-suspended Hela cells on a fibroblast-layered nanofibrous membrane via inkjet printing	[99]
Inkjet	n.s.	2	2	T, pH	n.s.	n.s.	n.s.	n.s.	n.s.	piezocrystal	-	n.s.	The behaviour of chondrocytes and osteoblasts to hyaluronic acid and type I collagen hydrogels	[100]
Inkjet	10× DMEM, H_2_O, NaOH	1.25	0.25	T, pH	n.s.	n.s.	n.s.	n.s.	n.s.	n.s.	-	n.s.	Evaluation and comparison of 12 hydrogels to determine suitability for bioprinting	[101]
Inkjet	n.s.	0.3	1	T, pH	n.s.	30	n.s.	50	300	microvalve	-	n.s.	A freeform and cell-friendly drop-on-demand bioprinting strategy for creating corneal stromal 3D models as suitable implants	[102]
Inkjet	1× PBS	2.05	1	T, pH (NaHCO_3_)	On ice	5–40	5–40	13.8	150	microvalve	-	7.6	Freeform fabrication technique, based on direct cell dispensing, implemented using a robotic platform that prints collagen hydrogel precursor, fibroblasts and keratinocytes.	[17]
Inkjet	1× PBS	1.12	1, 3	T, pH (NaHCO_3_)	On ice	5–40	5–40	13.8	150	microvalve	-	7.6	A direct cell printing technique to pattern neural cells in a three-dimensional (3D) multilayered collagen gel	[103]
Inkjet	1× PBS	2.23	1	T, pH (NaHCO_3_)	On ice	5–40	5–40	11–13.8	150	microvalve	-	n.s.	3D direct printing technique to construct hydrogel scaffolds containing fluidic channels	[76]
Inkjet	1× PBS	3	0.5-5	T, pH (NaHCO_3_)	On ice	n.s.	n.s.	18	n.s.	microvalve	-	52	The potential of 3D bioprinting for tissue engineering using human skin as a prototypical example	[21]
Inkjet	10× PBS, H_2_O, NaOH, FBS, DMEM	n.s.	1, 5, 10	T, pH	On ice	n.s.	n.s.	34.4	n.s.	piezocrystal	-	6	A bioprinter that can be used to print 3D patches of smooth muscle cells encapsulated within collagen	[104]
Inkjet	NaOH	3	2	T, pH	n.s.	n.s.	n.s.	n.s.	n.s.	piezocrystal	-	n.s.	Direct comparisons between commercially available hydrogels in the context of their cytocompatibility toward MSCs and their physicochemical parameters	[105]
Inkjet	NaOH	3	n.s.	T, pH	n.s.	n.s.	n.s.	n.s.	n.s.	piezocrystal	-	n.s.	Comparison of popular commercially available bioprinting hydrogels in the context of their physicochemical and other parameters	[106]
Inkjet	1× PBS, NaOH	1.3	0.06	T, pH	On ice	n.s.	37	n.s.	n.s.	n.s.	-	n.s.	The use of this system for the study of tumorigenesis and microenvironmental redirection of breast cancer cells	[107]
Inkjet	n.s.	n.s.	0.5	T, pH	On ice	n.s.	n.s.	n.s.	n.s.	n.s.	-	100	The proliferation of primary rat bladder smooth muscle cells in printed cell-laden collagen droplets	[108]
LaBP	10× PBS, NaOH	n.s.	35	n.s.	-	-	-	-	-	-	n.s.	n.s.	Fully cellularized skin substitute made of fibroblasts and keratinocytes on top of a stabilizing matrix (Matriderm®)	[109]
LaBP	10× DMEM/Ham’s F12, NaHCO3	3	35	T, pH	-	-	-	-	-	-	n.s.	0.1–1	The 3D arrangement of vital cells by LaBP as multicellular grafts analogous to native archetype and the formation of tissue by these cells	[110]
LaBP	n.s.	2	120	T, pH	-	-	-	-	-	-	50	n.s.	In situ printing of mesenchymal stromal cells, associated with collagen and nano-hydroxyapatite, in order to favour bone regeneration, in a calvaria defect model in mice	[111]
LaBP	10× PBS, NaOH, EDTA, thrombin, TBS	3	30	n.s.	-	-	-	-	-	-	18	n.s.	3D cornea-mimicking tissues using human stem cells and laser-assisted bioprinting	[112]
LaBP	n.s.	2	100	T, pH	-	-	-	-	-	-	n.s.	n.s.	The effect of distance between printed cell islets and the influence of coprinted mesenchymal cells on migration	[113]
LaBP	1× DMEM	2	70	T, pH	-	-	-	-	-	-	26	n.s.	A microvascular network following a defined pattern and its preservation while preparing the surface to print another layer of endothelial cells	[114]
LaBP	n.s.	2	n.s.	T, pH	-	-	-	-	-	-	28	n.s.	Organizing endothelial cells in situ, in a mouse calvaria bone defect, to generate a prevascularization with a defined architecture, and promote in vivo bone regeneration	[115]

## Data Availability

Not applicable.
